# Tapped-inductor bi-directional Cuk converter with high step-up/down conversion ratio and its optimum design

**DOI:** 10.1038/s41598-022-17801-z

**Published:** 2022-08-12

**Authors:** Hongxing Chen, Wei-ming Lin, Wen-ran Liu, Wei He

**Affiliations:** 1 Fujian Engineering Research Center of Safety Control for Ship Intelligent Navigation, College of Physics and Electronic Information Engineering, Minjiang University, Fuzhou, Fujian China; 2 Fujian Key Laboratory of New Energy Generation and Power Conversion, Fuzhou University, Fuzhou, 350108 China

**Keywords:** Engineering, Electrical and electronic engineering

## Abstract

A bidirectional DC–DC converter is required for an energy storage system. High efficiency and a high step-up and step-down conversion ratio are the development trends. In this research, a series of bidirectional high-gain Cuk circuits was derived by combining tapped inductors and bidirectional Cuk. After analyzing and comparing the characteristics of each circuit, a bidirectional high-gain Cuk circuit with a tapped-inductor (reverse coupling) was proposed. The proposed converter has a simple structure and a high voltage gain in both the step-down (Buck) and step-up (Boost) operation modes. The voltage stress of S_2_ was low. The voltage stress of S_1_ was high, however, and this is a disadvantage of the proposed converter. The proposed circuit’s characteristics were thoroughly examined, including the voltage gain characteristics and the design of the main parameters. We established a power loss model of the new topology, and the tapped-inductor turn ratio was optimized for high efficiency. Finally, a 400 W experimental implementation of the converter was shown to achieve efficiencies of 93.5% and 92.4% in the step-up and step-down modes, respectively. These findings verified the validity of the proposed circuit’s theoretical analysis.

## Introduction

Because of the scarcity of fossil fuels and serious environmental issues in recent years, significant effort has been focused on the development of environmentally friendly distributed generation (DG) technologies^1^. Renewable energy, however, does not produce consistent energy because of weather conditions. Energy storage is required to provide stable power^2^. Furthermore, the voltage of a storage battery is typically low, in the 12–48 V range, whereas the voltage of a DC bus is 400 V or higher to meet the requirements of an inverter or AC grid^3^. As a result, for energy storage systems to connect a low-voltage battery to a high-voltage DC bus, a bidirectional DC–DC converter with a high step-up/step-down voltage conversion ratio is required^4^. Furthermore, these converters have been researched extensively for a wide range of industrial applications, including uninterruptible power supply systems, electric vehicles, and aviation power supplies^5^. The traditional buck-boost converter can provide a high voltage gain with a large duty ratio, which will cause considerable conduction losses because of the large current ripples. Additionally, several bidirectional DC–DC converters based on isolated topologies have been presented in the literature. These topologies require a transformer and a high number of switching devices, which increases the cost and the switching losses, in addition to requiring more complicated control schemes.

Many bidirectional DC–DC converters with a high step-up/step-down conversion ratio have been proposed to improve the voltage gain and efficiency of a converter. The cascade method was used in reference ^6^ to broaden the ratio range of a bidirectional converter whose gain was calculated by multiplying the gains of each level converter. The efficiency was low, however, because of the cascade, and there was an issue of instability. The proposed converter in reference ^7^ improved a bidirectional DC-DC converter’s conversion ratio by connecting the low-voltage side in parallel and the high-voltage side in a series, but the structure of the converter was complex. Some appealing solutions, such as switched capacitors^8,9^, switched inductors^10^, and coupled inductors^11^, have been introduced for a basic bidirectional DC/DC converter to increase the voltage conversion ratio. The proposed bidirectional bridge modular switched-capacitor-based resonant DC–DC converter achieved a high step-up/step-down conversion ratio through a switched capacitor unit^8^. However, it employed a large number of switches, and the voltage and current stresses on the switches were high due to resonance. As a result, although the circuit proposed in ^9^ reduced the number of switches, its conversion ratio range was limited. Reference ^10^ employed the coupled-inductor technique to build a bidirectional DC–DC converter with a high step-up/step-down voltage gain. The current ripple was large because the current waveform on the low-voltage side of the topology was a square wave. In addition, reference ^11^ discussed nonisolated bidirectional DC–DC converters based on dual-coupled inductors, which could achieve a high voltage gain and reduced switch voltage stresses by connecting the secondary windings of two coupled inductors in series. However, it necessitated a complex control.

In summary, these isolated converter structures usually have too many switches, so the conduction losses of the switches were very high. Additionally, the practical implementation is complicated and expensive. The existing nonisolated high-gain circuits are mainly switch capacitor converters and coupled inductor converters. The drawbacks of a switch capacitor converter include the switching loss and the current stress. The drawbacks of a coupled inductor converter include the complex circuit structure and the leakage inductance that results in spikes that need to be suppressed using snubber circuits.

Cuk converters are gaining popularity because the input and output inductors reduce electromagnetic interference problems and the output ripple is small^12^. In this research, tapped inductance and bidirectional Cuk are combined to create a series of bidirectional high-gain Cuk circuits. After analyzing and comparing the characteristics of each circuit, a bidirectional high-gain Cuk circuit with a tapped-inductor (reverse coupling) is proposed. The proposed converter has a simple structure and high voltage gain in both the step-down (Buck) and step-up (Boost) operation modes. The proposed circuit’s characteristics, including the voltage gain characteristics and the design of the main parameters, are thoroughly examined. Based on this examination, we established a power loss model of the new topology, and the tapped-inductor turn ratio was optimized for high efficiency. Finally, a 400 W 48 V/400 V prototype was created to verify the validity of the proposed circuit’s theoretical analysis.

## A tapped-inductor bidirectional Cuk

**Figure 1:**
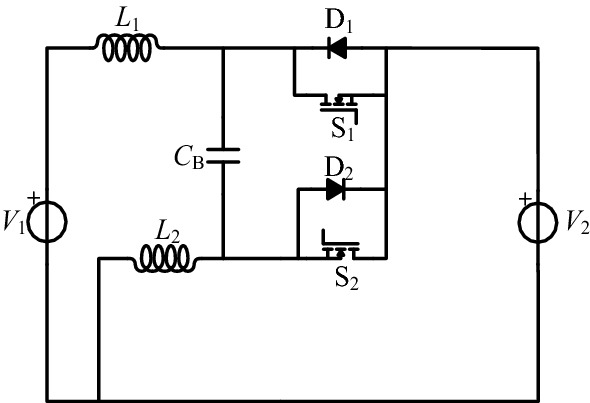
Bidirectional Cuk circuit.

The bidirectional Cuk circuit features low input and output ripple and low EMI interference, and the circuit diagram is shown in Fig. 1. Because of the influence of the parasitic parameters, the voltage gain of this circuit is limited, and it is not suitable for occasions with a large voltage transformation ratio. Therefore, a series of bidirectional high-gain Cuk circuits is created by combining tapped inductance and bidirectional Cuk to increase the voltage gain of bidirectional Cuk.

**Figure 2:**
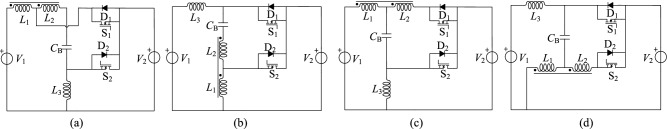
Bidirectional high-gain Cuk circuits are formed by the same-direction coupling, (**a**) S_1_-tap, (**b**) S_2_-tap, (**c**) *C*_B_-tap 1, (**d**) *C*_B_-tap 2.

**Figure 3:**
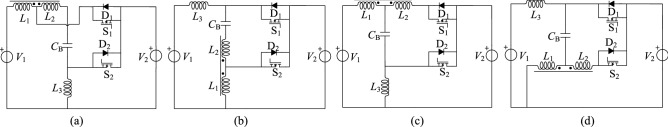
Bidirectional high-gain Cuk circuits are formed by the reverse-direction coupling, (**a**) S_1_-tap, (**b**) S_2_-tap, (**c**) *C*_B_-tap 1, (**d**) *C*_B_-tap 2.

The proposed series of circuits use coupled inductors to replace the inductors *L*_1_ or *L*_2_ in Fig. 1. Because of the different connection methods of the coupled inductor, four different circuits can be derived. Additionally, because the coupled inductor has two coupling modes (i.e., same-direction coupling and reverse-direction coupling), a total of eight circuits can be derived, as shown in Figs. 2 and 3. These converters with tapped inductors are formed by the same-direction coupling shown in Fig. 2. The reverse-direction coupling is shown in Fig. 3. The tapped inductor is composed of *L*_1_ with the number of turns *N*_1_ and *L*_2_ with the number of turns *N*_2_, where the tap ratio is *n* = *N*_2_: *N*_1_. Furthermore, D_1_ is the parasitic body diode of S_1_ and D_2_ is the parasitic body diode of S_2_. The same-direction coupling means that the currents all flow from the same-named end of the inductor and vice versa.

The voltage gain M of these converters versus the duty ratio D and the turn ratio *n* is obtained for the continuous current mode (CCM) mode by analyzing the working principles of the previously noted circuits, as shown in Table 1. S_1_-tap means that the inductor *L*_1_ of the bidirectional Cuk circuit is replaced by the tap inductor *L*_t_, and the common terminal of the tapped inductor is connected to S_1_, as shown in Fig. 2a and Fig. 3a. S_2_-tap means that the inductor *L*_2_ of the bidirectional Cuk circuit is replaced by the tap inductor *L*_t_, and the common terminal of the tapped inductor is connected to S_2_, as shown in Figs. 2b and 3b. *C*_B_-tap 1 means that the inductor *L*_1_ of the bidirectional Cuk circuit is replaced by the tap inductor *L*_t_, and the common terminal of the tapped inductor is connected to *C*_B_, as shown in Figs. 2c and 3c. *C*_B_-tap 2 means that the inductor *L*_2_ of the bidirectional Cuk circuit is replaced by the tap inductor *L*_t_, and the common terminal of the tapped inductor is connected to *C*_B_, as shown in Figs. 2d and 3d.

**Table 1:** The voltage gain M versus duty ratio D and the turn ratio *n.*

Circuit	S_1_-tap (same coupling)		S_2_-tap (same coupling)		C_B_-tap 1 (same coupling)		C_B_-tap 2 (same coupling)	
	Buck (V_2_/V_1_)	Boost (V_1_/V_2_)	Buck (V_2_/V_1_)	Boost (V_1_/V_2_)	Buck (V_2_/V_1_)	Boost (V_1_/V_2_)	Buck (V_2_/V_1_)	Boost (V_1_/V_2_)
Voltage gain	$\frac{(1 + n)D}{{1 - n(1 - D)}}$	$\frac{1 - n(1 - D)}{{(1 + n)(1 - D)}}$	$\frac{D}{1 + n(1 - D)}$	$\frac{1 + nD}{{1 - D}}$	$\frac{D}{1 + n(1 - D)}$	$\frac{1 + nD}{{1 - D}}$	$\frac{(1 + n)D}{{1 - n(1 - D)}}$	$\frac{1 - n(1 - D)}{{(1 + n)(1 - D)}}$

Circuit	S_1_-tap (reverse coupling)		S_2_-tap (reverse coupling)		C_B_-tap 1 (reverse coupling)		C_B_-tap 2 (reverse coupling)	
	Buck (V_2_/V_1_)	Boost (V_1_/V_2_)	Buck (V_2_/V_1_)	Boost (V_1_/V_2_)	Buck (V_2_/V_1_)	Boost (V_1_/V_2_)	Buck (V_2_/V_1_)	Boost (V_1_/V_2_)
Voltage gain	$\frac{(1 - n)D}{{1 - nD}}$	$\frac{1 - n(1 - D)}{{(1 - n)(1 - D)}}$	$\frac{D}{1 - n(1 - D)}$	$\frac{1 - nD}{{1 - D}}$	$\frac{D}{1 - n(1 - D)}$	$\frac{1 - nD}{{1 - D}}$	$\frac{(1 - n)D}{{1 - nD}}$	$\frac{1 - n(1 - D)}{{(1 - n)(1 - D)}}$

**Figure 4:**
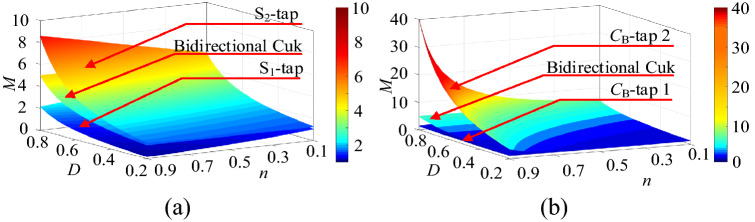
The voltage gain characteristic curves of the eight circuits in the step-up mode, (**a**) the same-direction coupling, (**b**) the reverse-direction coupling.

The corresponding curve can be drawn using the data from Table 1, as shown in Fig. 4. The voltage-gain characteristic curve of the circuits formed by same-direction coupling is shown in Fig. 4a. The curves of the S_1_-tap circuit and capacitor-tap circuit 2 are overlapped, and the curves of the S_2_-tap circuit and capacitor-tap circuit 1 are overlapped. As shown in Fig. 4a, the bidirectional Cuk circuit with the tapped inductor derived from S_2_-tap and capacitor-tap circuit 1 can achieve a high voltage gain. The voltage conversion ratio characteristic curve of the circuits formed by the reverse-direction coupling is shown in Fig. 4b. The curves of the S_1_-tap circuit and capacitor-tap circuit 2 are overlapped, and the curves of the S_2_-tap circuit and capacitor-tap circuit 1 are overlapped. As shown in Fig. 4b, the bidirectional Cuk circuit with the tapped inductor derived from S_1_-tap and capacitor-tap circuit 2 can achieve a high voltage gain.

**Figure 5:**
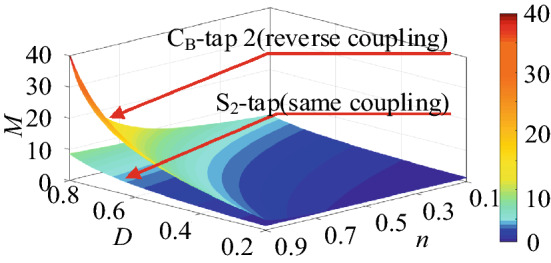
The voltage gain characteristic curves of the 4 circuits in step-up mode.

The voltage conversion ratio characteristic curves of the circuits in Figs. 3d and 2b are plotted, as shown in Fig. 5, to obtain the circuit with a larger step-up ratio from the previously noted circuits. As a result, it is determined that the circuit in Fig. 3d is the best of the previously noted circuits.

Because the analysis of these converters in the step-down mode is similar to the analysis in the step-up mode, it is not repeated here.

The feasibility analysis of the topologies’ large ratio is shown in Table 2 based on the preceding analysis. In the table, the term “inapplicable” means that the conversion ratio of this circuit is less than that of the bidirectional Cuk circuit, and the term “available” means that the conversion ratio of this circuit is greater than that of the bidirectional Cuk circuit.

**Table 2:** The feasibility analysis of the topologies’ large ratio.

Circuit		The feasibility analysis
The same coupling	S_1_-tap	Inapplicable
	S_2_-tap	Available
	C_B_-tap 1	Available
	C_B_-tap 2	Inapplicable
The reverse coupling	S_1_-tap	Available (high gain)
	S_2_-tap	Inapplicable
	C_B_-tap 1	Inapplicable
	C_B_-tap 2	Available (high gain)

## A tapped-inductor bidirectional high gain Cuk converter

### The circuit topology

**Figure 6:**
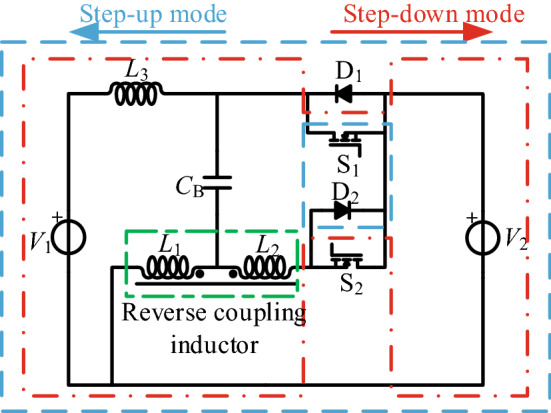
A reverse coupling tapped-inductor high gain bidirectional Cuk converter.

**Figure 7:**
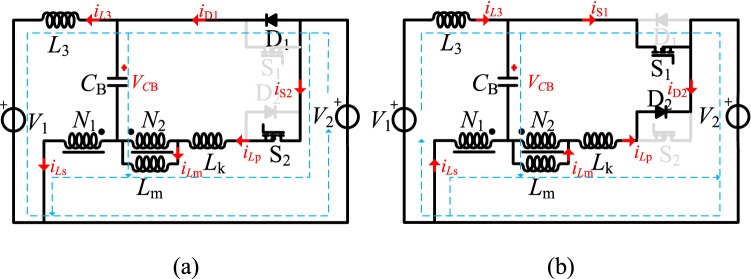
A reverse coupling tapped-inductor high gain bidirectional Cuk equivalent circuit, (**a**) step-up mode, (**b**) step-down mode.

According to this analysis, we proposed a tapped-inductor bidirectional Cuk converter with a high step-up/step-down conversion ratio, as shown in Fig. 6. The proposed converter is made up of the following components: the low-side voltage *V*_2_, the high-side voltage *V*_1_, the inductor *L*_3_, the tapped inductor *L*_t_, the capacitor *C*_B_, and the two switches S_1_–S_2_. The tapped inductor *L*_t_ is composed of *L*_1_ and *L*_2_ coupled in the opposite direction, and the turns of the inductor are *N*_1_ and *N*_2_(*N*_1_ > *N*_2_). The equivalent circuits of these stages are shown in Fig. 7.

The effective turn ratio of the tapped inductor is expressed as follows.

1 $$ \lambda  = \frac{{N_{1}  - N_{2} }}{{N_{1} }} $$

The coupling coefficient of the tapped inductor is:

2 $$ k = \frac{{L_{m} }}{{L_{m} + L_{k} }} $$

where *L*_m_ is the equivalent magnetizing inductance on the *N*_2_ side; and *L*_k_ is the leakage inductance on the *N*_2_ side.

### Operational principles

When using the proposed circuit in energy storage systems, the battery voltage *V*_2_ is on the low-voltage side and the DC-bus voltage *V*_1_ is on the high-voltage side. The proposed converter can operate in both step-up mode and step-down mode with bidirectional power flow. Reference ^13^ contains the operating principles and steady-state analysis. Hence, the simple results are discussed in the following, but the detailed analysis is not repeated.

**Figure 8:**
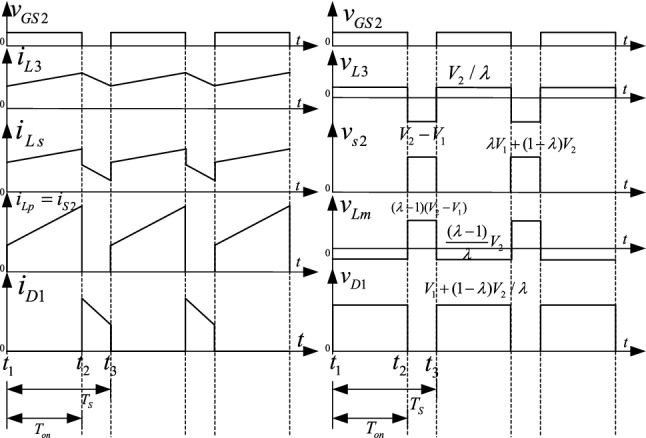
The main operation waveforms of the key components in the step-up mode.

**Figure 9:**
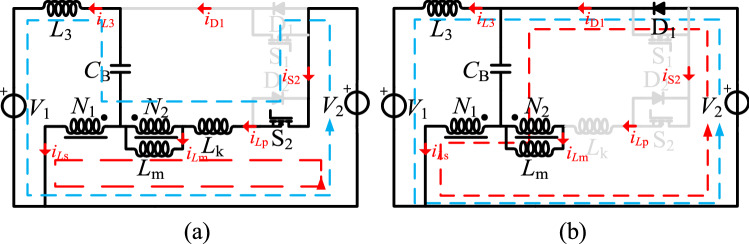
The equivalent circuit of the operation modes in the step-up mode, (**a**) S_2_ on, (**b**) S_2_ off.

As shown in Fig. 8, one switching period of the step-up mode has two switching stages. In Fig. 8, *v*_gs2_ is the driving signal of S_2_, the currents flowing through the *L*_1_, *L*_2_, and *L*_3_ inductors are *i*_*Ls*_, *i*_*Lp*_, and *i*_*L*3_, and *i*_D1_, *i*_S2_, and *i*_*C*B_ are the currents flowing through D_1_, S_2_, and *C*_B_. The equivalent circuits of these stages are shown in Fig. 9.

The gain of the proposed circuit in the step-up mode can be derived as follows.

3 $$ M_{up} = \frac{{V_{1} }}{{V_{2} }} = \frac{\lambda + (1 - \lambda )D}{{\lambda (1 - D)}} - \frac{D(1 - k)}{{\lambda (1 - D)}} $$

Ideally, the leakage inductor can be ignored, and the *M*_up_ can be derived as follows.

4 $$ M_{up} = \frac{{V_{1} }}{{V_{2} }} = \frac{\lambda + (1 - \lambda )D}{{\lambda (1 - D)}} $$

where *M*_up_ is the step-up conversion ratio of the proposed converter and *D* is the duty cycle of S_2_.

**Figure 10:**
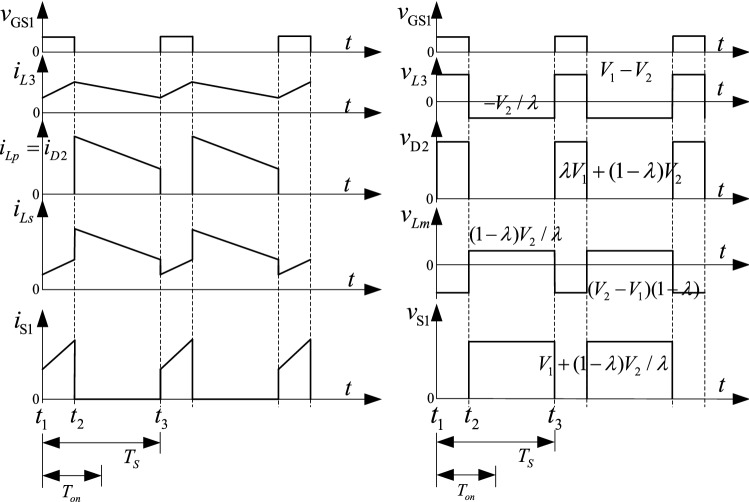
The main operation waveforms of the key components in the step-down mode.

**Figure 11:**
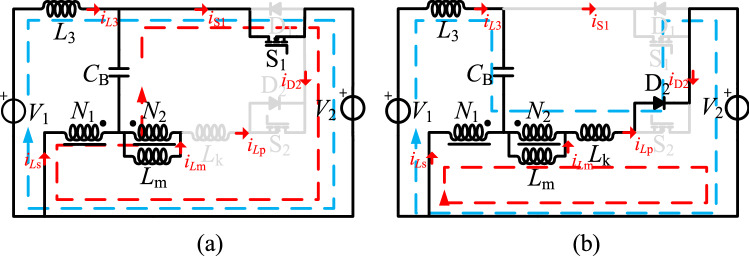
The equivalent circuit of the operation modes in the step-down mode, (**a**) S_1_ on, (**b**) S_1_ off.

As shown in Fig. 10, one switching period of the step-down mode has two switching stages. In Fig. 10, *v*_gs1_ is the driving signal of S_1_, the currents flowing through the *L*_1_, *L*_2_, and *L*_3_ inductors are *i*_*L*1_, *i*_*L*2_, and *i*_*L*3_, and *i*_D2_, *i*_S1_, and *i*_*C*B_ are the currents flowing through D_2_, S_1_, and *C*_B_. The equivalent circuits of these stages are shown in Fig. 11.

The gain of the proposed circuit in the step-down mode can be derived as follows.

5 $$ M_{{{\varvec{down}}}} = \frac{{V_{2} }}{{V_{1} }} = \frac{\lambda D}{{1 + (\lambda - 1)D}} + \frac{\lambda D(1 - D)(1 - k)}{{(1 + \lambda D - D)(k + \lambda D - kD)}} $$

Ideally, the leakage inductor can be ignored, and *M*_down_ can be derived as follows.

6 $$ M_{{{\varvec{down}}}} = \frac{{V_{2} }}{{V_{1} }} = \frac{\lambda D}{{1 + (\lambda - 1)D}} $$

where *M*_down_ is the step-down conversion ratio of the proposed converter and *D* is the duty cycle of S_1_.

### Comparison analysis of the proposed converter

The characteristic comparison of the proposed converter with the counterparts is shown in Table 3 (*NS* is the number of power switches, *N*CI is the number of coupled inductors, *N*I is the number of inductors, and *N*C is the number of capacitors). The conventional buck/boost converter can achieve bidirectional power flows while employing the fewest number of power switches, but the converter’s conversion ratio range is limited. The converter in reference ^14^ has a high step-up/step-down conversion ratio, but it is complex and inefficient. Compared with the converters in reference ^14^, the converter’s efficiency in reference ^15^ has been improved by using soft switching technology, but the circuit structure is still complex. It can be seen that the proposed converter achieves a high and wide voltage-gain range by employing two power switches. Additionally, it has a simple structure.

**Table 3:** Characteristic comparison of the proposed converter with the main competitors.

Bidirectional converter	Max. voltage stress of switches	Efficiency	Structural complexity	*N*_S_	*N*_CI_	*N*_I_	*N*_C_	Voltage gain	
								Step-up	Step-down
Buck/boost converter	*V*_H_	–	Simple	2	–	1	2	$\frac{1}{1 - D}$	*D*
The converter in ^14^	*V*_H_	88.9–92.3% (250 W)	Complex	4	1	–	3	$\frac{1}{{\left( {1 - D} \right)^{2} }}$	*D*^2^
The converter in ^15^	$\frac{1 + n}{{2 + n}} \cdot V_{H}$	88.7–94.2% (400 W)	Complex	4	1	1	5	$\frac{2 + n}{{1 - D}}$	$\frac{D}{2 + n}$
Proposed converter	$\frac{1}{1 + (1 - D)n} \cdot V_{H}$	89.3–93.5% (400 W)	Simple	2	1	1	2	$\frac{1 - n(1 - D)}{{(1 - n)(1 - D)}}$	$\frac{(1 - n)D}{{1 - nD}}$

### The feasibly control strategy for the proposed converter

**Figure 12:**
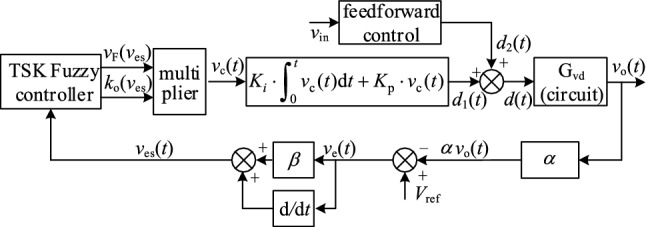
The improved fuzzy control schematic.

To improve the dynamic performance and antidisturbance ability of the proposed converter, we proposed an improved fuzzy control strategy based on the Takagi–Sugeno-Kang fuzzy control technique, as shown in Fig. 12. The operating principle and a detailed analysis of the control strategy can be obtained from reference ^13^. Therefore, the detailed analysis is not repeated in this paper.

## Analysis and design of the key parameters

### Optimized design of turn ratio

#### Power loss model

A power loss model of the new topology is established in the step-up mode. The loss of the proposed converter is composed of the losses of S_2_, *L*_t_, *L*_3_, and D_1_. The specific analysis is given as follows.

1. The loss of S_2_.
  The conduction loss is expressed as follows
  7 $$ P_{con\_S} = I_{rms\_S}^{2} \cdot R_{ds(on)\_S} $$
  where *I*_rms_S2_ is the effective value of the current across S_2_, and *R*_ds(on)_ is the forward conduction resistance of S_2_ at a certain temperature, which can be estimated from the datasheet and the ambient temperature.
  The switching loss is found as follows
  8 $$ P_{sw} = \frac{1}{2} \cdot f_{s} V_{ds} \cdot \left[ \begin{gathered} I_{d01} \cdot (t_{ri} + t_{fv} ) \hfill \\ { + }I_{d02} \cdot (t_{rv} + t_{fi} ) \hfill \\ \end{gathered} \right] $$
  where *t*_*ri*_, *t*_*fv*_, *t*_*r*_, and *t*_*fi*_ are the equivalent times of the four phases with the loss during the switching process, which can be calculated from the datasheet.
  The loss of the equivalent output capacitance of S_2_ is found as follows:
  9 $$ P_{{{\text{Co}} \_S}} = \frac{1}{2} \cdot C_{oss} \cdot V_{ds\_S2}^{2} \cdot f_{S} $$
  Therefore, the overall loss of S_2_ is given by the following:
  10 $$ P_{s} = P_{con\_S} + P_{sw} + P_{{{\text{Co}} \_S}} $$
2. The loss of D_1_
  11 $$ P_{D} = V_{F} \cdot I_{D} + V_{off\_D} \cdot Q_{rr} \cdot f_{S} $$
  where *V*_F_ is the forward voltage drop of D_1_, *I*_D_ is the average value of the current across D_1_, *V*_*off*_D_ is the reverse voltage of D_1_, and Q_rr_ is the reverse recovery charge of D_1_.
3. The loss of the inductor
  The core loss is found as follows:
  12 $$ P_{core} = f_{s} \cdot Kf_{eq}^{{{{\alpha - 1}}}} B^{\beta } \left( {C_{0} + C_{1} T + C_{2} T^{2} } \right) $$
  13 $$ f_{eq} (D) = \frac{2}{{B^{2} \pi^{2} }}\int\limits_{0}^{T} {(\frac{dB}{{dt}})^{2} dt = } \frac{{2f_{s} }}{{\pi^{2} D(1 - D)}} $$

The winding loss is found as follows

14 $$ P_{winding} = I_{L\_rms}^{2} \cdot R_{dc} $$

Therefore, the overall loss of inductor is given by the following:

15 $$ P_{core} = P_{core} \cdot V_{core} + P_{winding} $$

where the parameters *K*, α, β, *C*_0_, *C*_1_, and *C*_2_ can be obtained from the datasheet provided by the core manufacturer; *T* is the operating temperature of the magnetic core; *V*_core_ is the volume of the magnetic core; *I*_L_rms_ is the effective value of the current through the inductor; and *R*_dc_ is the equivalent resistance of the inductor.

The power loss models of *L*_t_ and *L*_3_ are similar to each other. Therefore, the description of the power loss model of *L*_t_ is not repeated here.

To summarize, the overall loss of the proposed converter in the step-up mode is given by the following:

16 $$ P_{total\_up} = P_{S2} + P_{D1} + P_{L3} + P_{Lt} $$

Hence, the efficiency of the proposed converter in the step-up mode is given as follows

17 $$ \eta_{up} = \frac{{P_{o} }}{{P_{o} + P_{total\_up} }} $$

Similarly, the overall loss of the proposed converter in the step-down mode is given by the following:

18 $$ P_{total\_down} = P_{S1} + P_{D2} + P_{L3} + P_{Lt} $$

Hence, the efficiency of the proposed converter in the step-down mode is given as follows

19 $$ \eta_{down} = \frac{{P_{o} }}{{P_{o} + P_{total\_down} }} $$

#### The optimization selection of turn ratio

The loss characteristics of the proposed circuit are analyzed using Mathcad and the power loss model from the previous section. The following are the converter’s main simulation parameters: *V*_2_ = 48 V, *V*_1_ = 400 V, *P*_o_ = 400 W, *L*_3_ = 1.5 mH, *L*_1_ = 0.9 mH, switching frequency: *fs* = 50 kHz.

**Figure 13:**
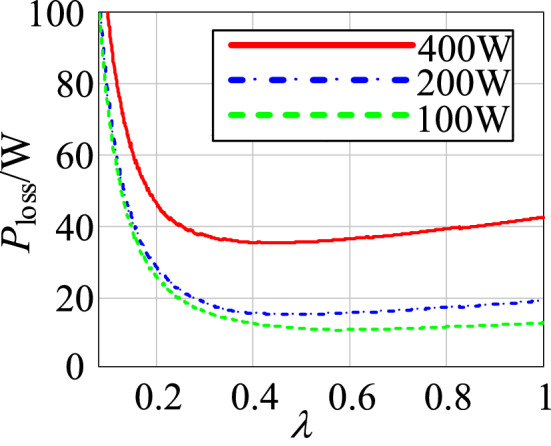
The curves between the loss of the proposed circuit and the turn ratio under different loads.

According to Formula (16), the curves for the loss of the proposed circuit and the turn ratio under different loads can be drawn using Mathcad, as shown in Fig. 13.

From Fig. 13, when the output power is constant, the total loss of the circuit decreases at first and then increases as the turn ratio increases. As a result, a minimum loss point serves as the foundation for selecting the appropriate turn ratio in this research.

**Figure 14:**
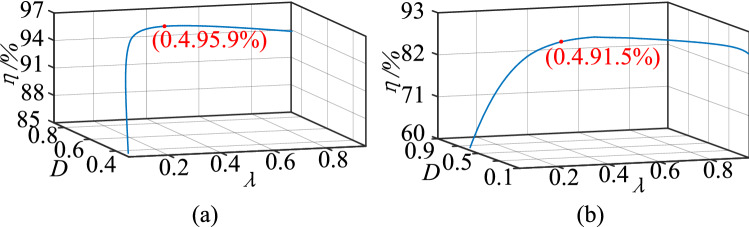
The calculation curve for λ, *D*, and the efficiency: (**a**) step-up mode, (**b**) step-down mode.

The calculation curve for the efficiency of the proposed circuit in the step-up mode can be drawn using Formula (17), as shown in Fig. 14a. Figure 14b depicts the calculation curve for the efficiency of the proposed circuit in the step-down mode, according to Formula (19).

As shown in Fig. 14, the circuit’s efficiency increases at first and then decreases as the turn ratio increases. There is a maximum level of efficiency. As a result, to achieve the expected output and high efficiency, an appropriate turn ratio and steady-state duty ratio should be chosen. The turn ratio should be around 0.4, and the duty cycle should be around 0.75, according to Fig. 14.

**Figure 15:**
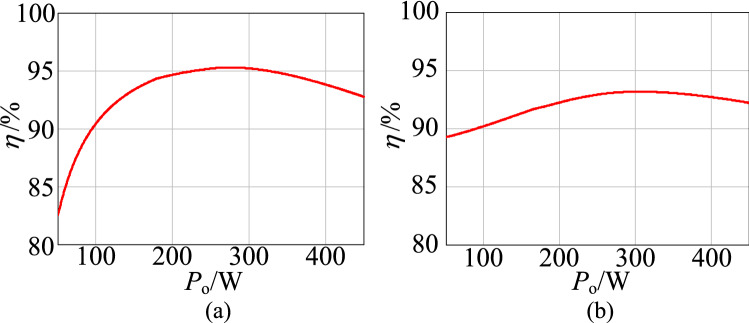
The calculation curve of the efficiency, (**a**)step-up mode, (**b**) step-down mode.

Given the possibility of errors during the design and winding processes, the best turn ratio is ${\lambda }_{opt}$ = 0.375–0.412. The efficiency calculation curves are shown in Fig. 15. When the proposed converter operates under rated conditions, the best turn ratio is ${\lambda }_{opt}$ = 0.394. Figure 15a depicts the efficiency curve in the step-up mode, and Fig. 15b shows the efficiency curve in the step-down mode.

### Other parameters design

#### The selection of the inductor

To ensure that the circuit works in CCM mode, the values of *L*_1_, *L*_2_, and *L*_3_ must be greater than the inductance value with critical continuity. These values are given as follows:

20 $$ L_{1} \ge \frac{1}{{\frac{{2\lambda f_{s} I_{1} }}{{(V_{1} - V_{2} )(1 - D)}} - \frac{1 - \lambda }{{L_{3} }}}} $$

21 $$ L_{2} = L_{1} (1 - \lambda )^{2} $$

22 $$ L_{3} \ge \frac{{V_{1} - V_{2} }}{{f_{{\text{s}}} (2 \cdot I_{L3} )}} = \frac{{V_{1} - V_{2} }}{{2I_{{1}} f_{{\text{s}}} }} $$

#### The selection of capacitor

The selection of the capacitor mainly includes consideration of the voltage stress and the voltage ripple within a certain range. The value of *C*_B_ is found as follows:

23 $$ C = \frac{{|i_{CB} | \cdot DT_{{\text{s}}} }}{{\Delta V_{C} }} $$

## Simulation and experimental verification

### Simulation results

We performed detailed simulations in Matlab/Simulink to verify the correctness of the aforementioned theoretical analysis. The proposed converter operation is verified at *V*_2_ = 48 V, *V*_1_ = 400 V, *P*_o_ = 400 W, *L*_3_ = 1.5 mH, *L*_1_ = 0.9 mH, *L*_2_ = 0.33 mH, *L*_k_ = 0.92 uH, *C*_B_ = 2.2 uF, *C*_o1_ = 47 uF, *C*_o2_ = 47 uF, and the switching frequency *f*s = 50 kHz.

**Figure 16:**
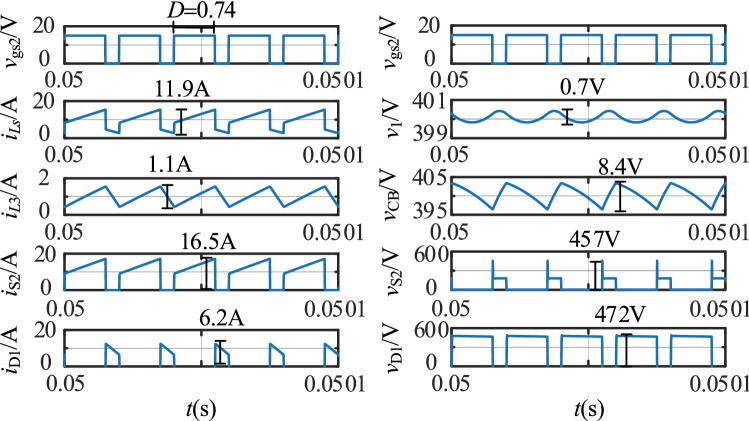
The stable waveforms of the key components in the step-up mode.

The simulation results in the step-up mode at full load are shown in Fig. 16. In Fig. 16, *v*_gs2_ is the driving signal for S_2_, the currents flowing through the *L*_1_, *L*_2_, and *L*_3_ inductors are *i*_*L*s_, *i*_*Lp*_, and *i*_*L*3_, and *i*_D1_, *i*_S2_, and *i*_*C*B_ are the currents flowing through D_1_, S_2_, and *C*_B_, respectively.

**Figure 17:**
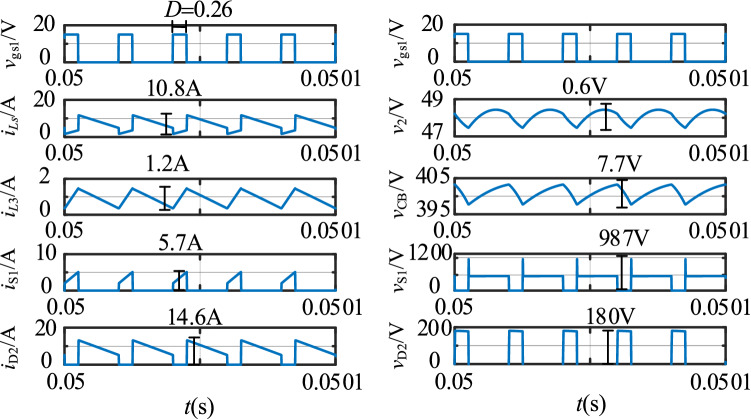
The stable waveforms of the key components in the step-down mode.

The simulation results in the step-down mode at full load are shown in Fig. 17. *v*_gs1_ is the driving signal for S_1_, the currents flowing through the *L*_1_, *L*_2_, and *L*_3_ inductors are *i*_*L*s_, *i*_*L*p_, and *i*_*L*3_, and *i*_D2_, *i*_S1_, and *i*_*C*B_ are the currents flowing through D_2_, S_1_, and *C*_B_ separately.

In the step-up mode, the output voltage is stable at 400 V, as shown in Fig. 16. The duty cycle of S_2_ is 0.74. The voltage stresses of S_2_ and D_1_ are 457 V and 472 V. Similarly, Fig. 17 shows that the output voltage is stable at 48 V in the step-down mode. The duty cycle of S_1_ is 0.26. The voltage stresses of S_1_ and D_2_ are 987 V and 180 V. The voltage and current spikes of S_1_, S_2_, and the inductor are caused by the leakage inductance of the coupled inductor. Thus, the results in Figs. 16 and 17 show that the simulation results closely match the theoretical analysis.

### Experimental results

To validate the theoretical analysis, we built a laboratory prototype of the proposed converter. First, based on typical applications, we selected the operating conditions of the proposed converter as *V*_2_ = 48 V, *V*_1_ = 400 V, and *P*_o_ = 400 W. Second, according to Formulas (20)–(23), *L*_3_ = 1.5 mH, *L*_1_ = 0.9 mH, *L*_2_ = 0.33 mH, *C*_B_ = 2.2 uF, *C*_o1_ = 100 uF, and *C*_o2_ = 100 uF. Then the voltage-current stress of the semiconductor device can be obtained by analyzing the specific operating principle of the converter. The voltage-current stress of S_1_ is as follows:

24 $$ \left\{ {\begin{array}{*{20}c} {v_{{{\varvec{S}}1.\max }} = V_{1} + \frac{1 - \lambda }{\lambda }V_{2} = \frac{1}{1 + (\lambda - 1)D}V_{1} } \\ {i_{{{\varvec{S}}1.\max }} = \frac{\lambda }{1 - (1 - \lambda )D}I_{2} + \frac{{D(V_{1} - V_{2} )(L_{1} + L_{3} )}}{{2f_{{\varvec{s}}} L_{1} L_{3} }}} \\ \end{array} } \right. $$

The voltage-current stress of S_2_ is as follows:

25 $$ \left\{ {\begin{array}{*{20}c} {v_{{{\varvec{S}}2.\max }} = \lambda V_{1} + (1 - \lambda )V_{2} = \frac{\lambda }{1 + (\lambda - 1)D}V_{1} } \\ {i_{{{\varvec{S}}2.\max }} = \frac{{I_{1} }}{\lambda (1 - D)} + \frac{{(1 - D)(V_{1} - V_{2} )(L_{1} + L_{3} )}}{{2\lambda f_{{\varvec{s}}} L_{1} L_{3} }}} \\ \end{array} } \right. $$

where *I*_1_ is the average value of the high-voltage side current and *I*_2_ is the average value of the low-voltage side current.

**Figure 18:**
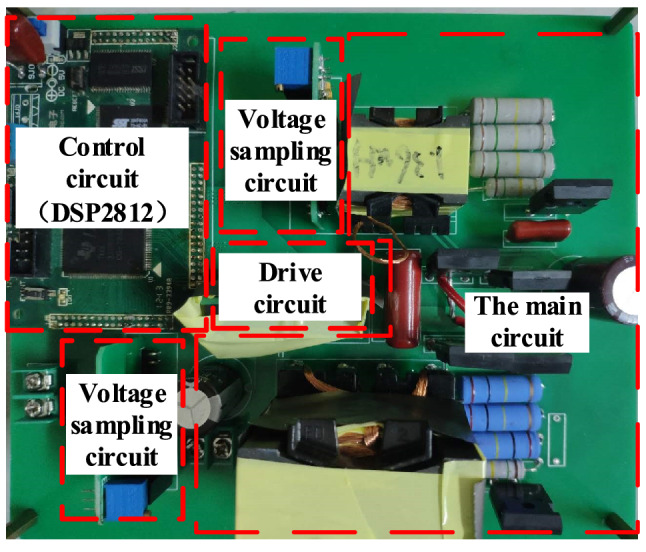
Prototype of the converters.

The maximum voltage and the current stress values of S_1_ and S_2_ are obtained by incorporating the corresponding parameters. Then, based on a certain margin, the specific type of switching tube that is required can be selected. The specific parameters of the proposed converter are listed in Table 4, and the prototype is shown in Fig. 18.

**Table 4:** The parameters of the proposed circuit.

Parameters	The proposed converter
*V*_1_/V	400 (250–430)
*V*_2_/V	48 (36–60)
*P*_o_/W	400
*L*_1_,*L*_*2*_/uH	900:346
*L*_*k*_/uH	0.92
*L*_3_/mH	1.5
*C*_B_	2.2 uF
*C*o	100 uF
S_1_	STP20N95K5
S_2_	TK39N60W
*f*_s_/kHz	50

**Figure 19:**
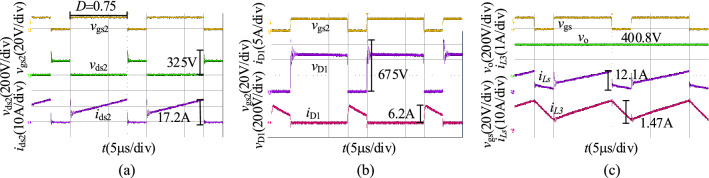
The experiment results of the proposed converter in the step-up mode when *v*_2_ = 48 V: (**a**) *v*_gs2_, *v*_ds2_, *i*_ds2_, (**b**) *v*_gs2_, *v*_D1_, *i*_D1_, (**c**) *v*_gs2_, *v*_1_, *i*_*L*1_, *i*_*L*3_.

When *v*_2_ = 48 V, we obtain the experimental results in the step-up mode at a full load as shown in Fig. 19. Figure 19a shows the waveforms of *v*_gs2_, *v*_ds2_, and *i*_ds2_, and the duty cycle of S_2_ is 0.75. The voltage stress of S_2_ is 325 V. Figure 19b shows the waveforms of *v*_gs2_, *v*_D1_, and *i*_D1_, and the voltage stress of D_1_ is 675 V. Figure 19c shows the waveforms of *v*_gs2_, *v*_1_, *i*_*L*1_, and *i*_*L*3_, and the output voltage of the proposed converter in step-up mode is 400.8 V.

**Figure 20:**
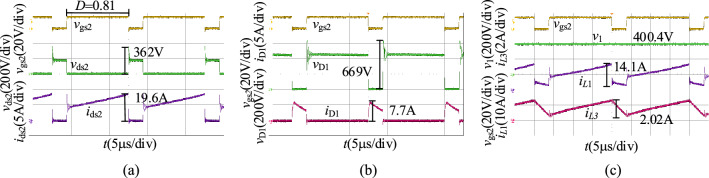
The experiment results of the proposed converter in the step-up mode when *v*_2_ = 36 V: (**a**) *v*_gs2_, *v*_ds2_, *i*_ds2_, (**b**) *v*_gs2_, *v*_D1_, *i*_D1_, (**c**) *v*_gs2_, *v*_1_, *i*_*L*1_, *i*_*L*3_.

When *v*_2_ = 36 V, we obtain the experimental results in the step-up mode at full load as shown in Fig. 20. As illustrated in Fig. 20, the duty cycle of S_2_ is 0.81, and the output voltage of the proposed converter in the step-up mode is 400.4 V. The voltage stresses of S_2_ and D_1_ are 362 V and 669 V.

**Figure 21:**
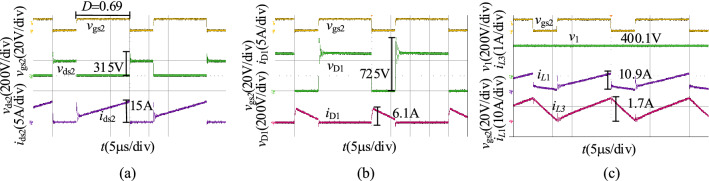
The experiment results of the proposed converter in the step-up mode when *v*_2_ = 60 V: (**a**) *v*_gs2_, *v*_ds2_, *i*_ds2_, (**b**) *v*_gs2_, *v*_D1_, *i*_D1_, (**c**) *v*_gs2_, *v*_1_, *i*_*L*1_, *i*_*L*3_.

When *v*_2_ = 60 V, we obtain the experimental results in the step-up mode at full load as shown in Fig. 21. As illustrated in Fig. 21, the duty cycle of S_2_ is 0.69, and the output voltage of the proposed converter in the step-up mode is 400.1 V. The voltage stresses of S_2_ and D_1_ are 315 V and 725 V.

Compare with the simulation results in Fig. 16, the experimental results in the step-up mode are consistent with it. Both of them are then consistent with the theoretical analysis. The voltage and current spikes are caused by the leakage inductance.

**Figure 22:**
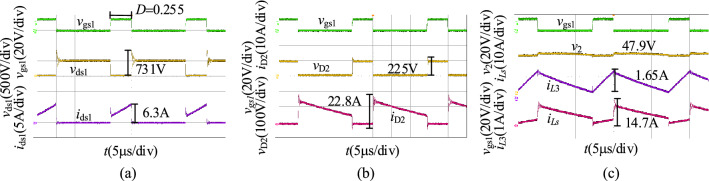
The experiment results of the proposed converter in the step-down mode when *v*_1_ = 400 V: (**a**) *v*_gs1_, *v*_ds1_, *i*_ds1_, (**b**) *v*_gs1_, *v*_D2_, *i*_D2_, (**c**) *v*_gs_, *v*_2_, *i*_*L*1_, *i*_*L*3_.

When *v*_1_ = 400 V, we obtain the experimental results in the step-up mode at full load as shown in Fig. 22. Figure 22a shows the waveforms of *v*_gs1_, *v*_ds1_, and *i*_ds1_, and the voltage stress of S_1_ is 731 V. Figure 22b shows the waveforms of *v*_gs1_, *v*_D2_, and *i*_D2_, and the voltage stress of D_2_ is 225 V. Figure 22c shows the waveforms of *v*_gs2_, *v*_o_, *i*_*L*1_, and *i*_*L*3_, and the output voltage of the proposed converter is 47.9 V.

**Figure 23:**
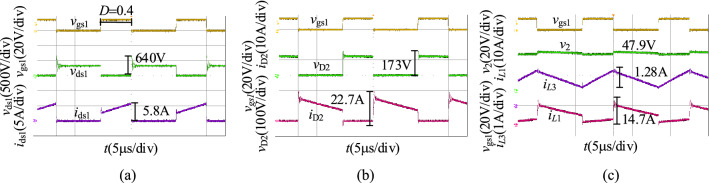
The experiment results of the proposed converter in the step-down mode when *v*_1_ = 250 V: (**a**) *v*_gs1_, *v*_ds1_, *i*_ds1_, (**b**) *v*_gs1_, *v*_D2_, *i*_D2_, (**c**) *v*_gs_, *v*_2_, *i*_*L*1_, *i*_*L*3_.

When *v*_1_ = 250 V, we obtain the experimental results in the step-down mode at full load as shown in Fig. 23. As illustrated in Fig. 23, the duty cycle of S_1_ is 0.4, and the output voltage of the proposed converter in the step-up mode is 47.9 V. The voltage stresses of S_1_ and D_2_ are 640 V and 173 V.

**Figure 24:**
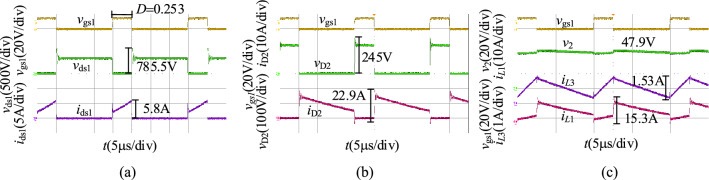
The experiment results of the proposed converter in the step-down mode when *v*_1_ = 430 V: (**a**) *v*_gs1_, *v*_ds1_, *i*_ds1_, (**b**) *v*_gs1_, *v*_D2_, *i*_D2_, (**c**) *v*_gs_, *v*_2_, *i*_*L*1_, *i*_*L*3_.

When *v*_1_ = 250 V, we obtain the experimental results in the step-down mode at full load as shown in Fig. 24. As illustrated in Fig. 24, the duty cycle of S_1_ is 0.253 and the output voltage of the proposed converter in the step-up mode is 47.9 V. The voltage stresses of S_1_ and D_2_ are 785.5 V and 245 V.

Similarly, compare with the simulation results in Fig. 17, the experimental results in the step-down mode are consistent with it. Both of them are then consistent with the theoretical analysis.

**Figure 25:**
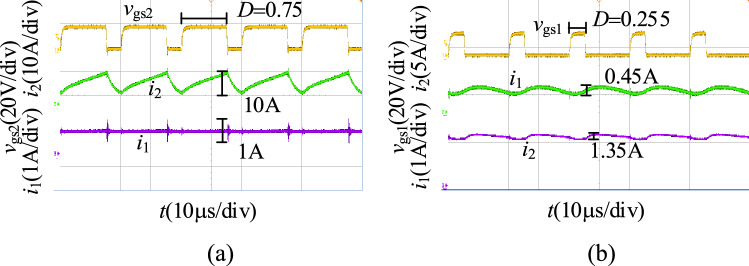
The input and output current waveforms of the proposed converter, (**a**) step-up mode, (**b**) step-down mode.

We obtain the input and output current waveforms in the step-up/step-down mode at full load as shown in Fig. 25. Figure 25a shows the current waveforms in the step-up mode, and Fig. 25b shows the current waveforms in the step-down mode. As illustrated in Fig. 25, the input and output current ripple of the proposed converter is low.

**Figure 26:**
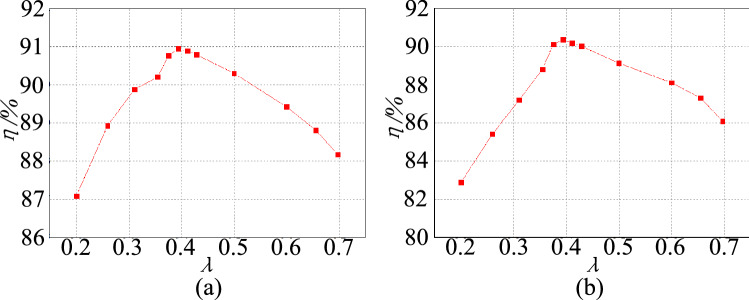
The efficiency curves of $\lambda$, (**a**) step-up mode, (**b**) step-down mode.

The measured efficiency curve of the experimental circuit in the step-up mode is shown in Fig. 26a. Figure 26b shows the measured efficiency curve of the experimental circuit in the step-down mode. Compare with Fig. 14, it can be seen that the proposed circuit’s measured efficiency curve agrees with the calculation curve. The trends of the curves are increased firstly and then decreased. Furthermore, because the actual total loss is not taken into account in the calculation, the maximum measured efficiency is less than the theoretical calculation value.

**Figure 27:**
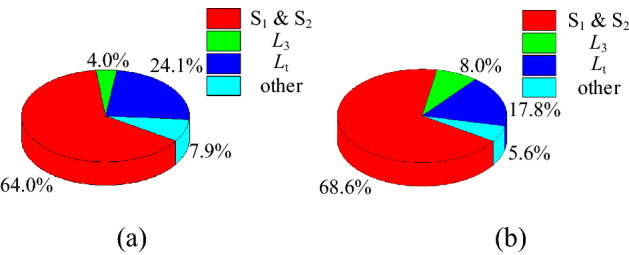
The experimental loss of the proposed converter, (**a**) step-up mode, (**b**) step-down mode.

When the proposed converter operates under rated conditions and the best turn ratio is ${\lambda }_{opt}$ = 0.394, we obtain the experimental loss of the proposed converter as shown in Fig. 27. As illustrated in Fig. 27, the loss is mainly concentrated on the switching and the coupled inductor in the step-up/step-down mode.

**Figure 28:**
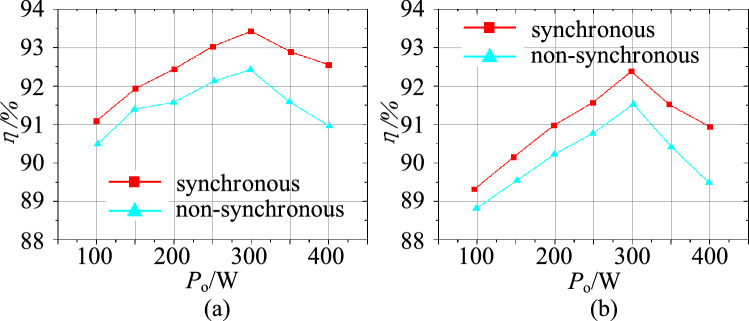
The efficiency curves with the load, (**a**) step-up mode, (**b**) step-down mode.

The conversion efficiency versus the output power in the step-up mode and step-down mode is plotted in Fig. 28.In the step-up mode, the maximum efficiency of the proposed converter is 93.5%. In the step-down mode, the proposed converter has a maximum efficiency of 92.2%.

Comparing Figs. 28 and 15, we found that the trends of the measured efficiency curve and the calculation curve ere consistent in the step-up/step-down mode. The trends increased at first and then decreased as the output power increased. Similarly, because the actual total loss was not taken into account, the maximum measured efficiency was less than the theoretical calculation value.

## Conclusion

The use of a tapped inductor in this research improved the bidirectional DC-DC converter’s conversion ratio and overcame the shortcomings of the nonisolated bidirectional DC–DC converter’s low conversion ratio. Furthermore, a series of bidirectional high-gain Cuk circuits was derived by summarizing and analyzing the various forms of the proposed coupled inductor. The best circuit was obtained by analyzing and comparing the characteristics of each circuit, and we proposed a bidirectional high-gain Cuk circuit with a capacitor-tapped inductor (reverse coupling). In both the step-down and step-up operation modes, this converter had a simple structure and a high voltage gain. Following this, the proposed circuit’s operational principles and characteristics were thoroughly examined. In addition, the efficiency of the proposed converter was improved further after the optimal selection of the coupled inductor’s turn ratio. Finally, we created a 400 W 48 V/400 V prototype to verify the validity of the proposed circuit’s theoretical analysis.

## Data Availability

The datasets of this study are available from the corresponding author on reasonable request.
